# A Case of Pelvic Schwannoma Presenting Prominent Eggshell-Like Calcification

**DOI:** 10.1155/2013/825078

**Published:** 2013-10-01

**Authors:** Takaaki Nakashima, Daisuke Tsurumaru, Yusuke Nishimuta, Mitsutoshi Miyasaka, Akihiro Nishie, Hiroshi Honda

**Affiliations:** Department of Clinical Radiology, Graduate School of Medical Sciences, Kyushu University, 3-1-1 Maidashi, Higashi-ku, Fukuoka City 812-8582, Japan

## Abstract

Pelvic schwannoma typically forms a large, well-circumscribed mass in the retroperitoneum or presacral area and frequently undergoes cystic degeneration. It appears as a well-demarcated round or oval mass, often showing prominent cystic degeneration and calcification. Characteristics of these calcifications are punctate, mottled, or curvilinear and are seen along the walls of the mass. Herein, we describe a case of schwannoma presenting a huge pelvic mass with unique eggshell-like calcification.

## 1. Introduction

Schwannomas (neurilemmomas) are benign neurogenic tumors originating in Schwann cells of the nerve sheath. They generally occur in the head and neck and extremities but rarely in the pelvis or retroperitoneum [1]. Retroperitoneal and pelvic schwannomas typically form large, well-circumscribed masses in the retroperitoneum or presacral area and frequently undergo cystic degeneration [2]. We report a case of pelvic schwannoma presenting prominent calcification.

## 2. Case Presentation

A 68-year-old man presented with anal pain for one month and underwent examination in a clinic. A giant pelvic mass was detected on imaging examinations, and he was admitted to our hospital for closer examination. He had no history of previous malignancy. On admission, he had only anal pain and no other symptoms. On rectal examination, a hard fixed mass was felt on the posterior wall of the rectum. No abnormal neurological signs were found. His blood test and tumor markers including CEA, CA19-9, and soluble IL-2 receptor were within normal limits.

**Figure 1:**
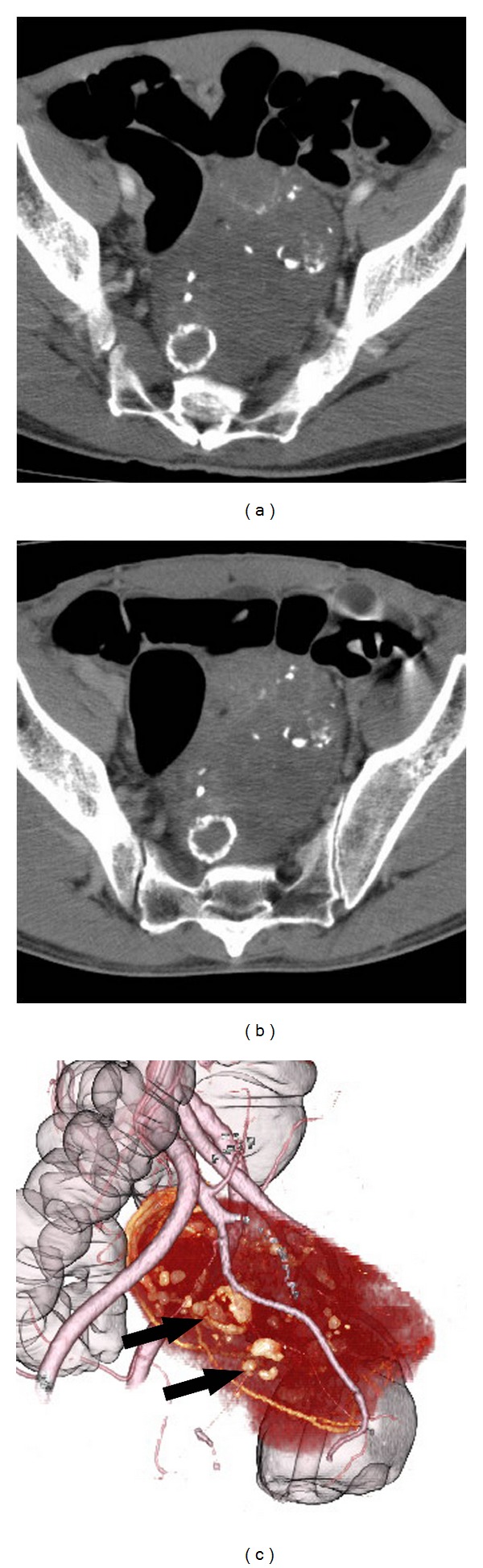
(a) Contrast-enhanced CT shows a low-density mass containing coarse and round calcifications in early phase (40 s). Cystic lesions are not evident. (b) The mass is slightly enhanced in delayed phase (180 s). (c) 3D-CT shows eggshell-like calcifications within the tumor (arrows).

**Figure 2:**
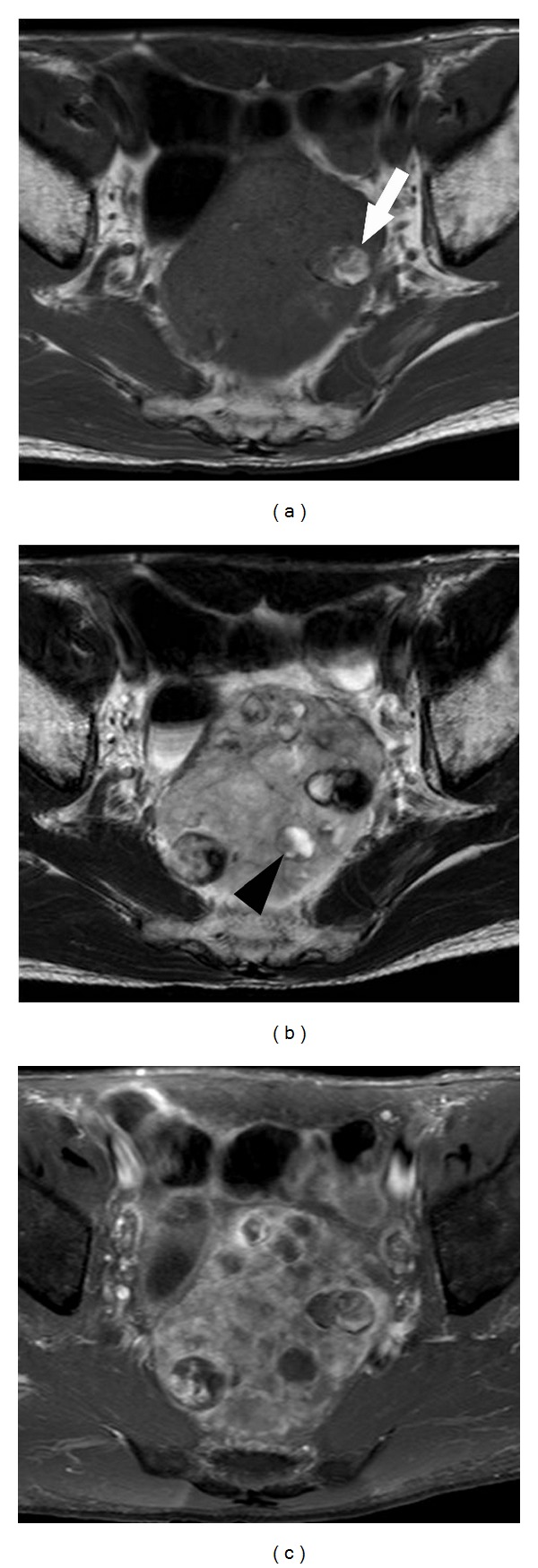
(a) T1-weighted MR image shows a well-defined hypointensity mass with focal high-intensity area (arrow). (b) T2-weighted image shows heterogeneous hyperintensity mass with spotty hyperintensity area (arrowhead). (c) Contrast-enhanced image shows slight enhancement in solid component of the tumor.

Contrast-enhanced computed tomography (CT) was performed using nonionic contrast material (Iopamiron 370; Bayer Health Care) and showed a well-defined hypoattenuation tumor in the pelvis measuring 87 × 65 mm in the transaxial diameter. The tumor was located close to the rectum, anterior to the sacrum. There were multiple, spotted, and oval eggshell-like calcifications within the tumor. Cystic change was not evident on CT. The tumor parenchyma revealed hypoattenuation in the early phase (40 s after administration of contrast agent) and was slightly enhanced in the delayed (240 s) phase (Figure 1). Magnetic resonance imaging (MRI) revealed a mass with heterogeneous low signal intensity on T1-weighted image and high signal intensity on T2-weighted image. There were high signal intensity areas indicating hemorrhage and signal voids corresponding to calcifications on the T1-weighted image and focal high signal intensity areas indicating myxoid or cystic degeneration on the T2-weighted image (Figure 2). A colonoscopy showed only luminal narrowing with no mucosal change in the rectum. Endoscopic ultrasound showed a huge hypoechoic mass with multiple hyperechoic rims indicating calcifications located in the extracolorectal region. These imaging features indicated the high possibility of a benign tumor. However, surgery was planned because the large pelvic tumor presented anal symptoms. Endoscopic ultrasound-guided fine needle aspiration biopsy (EUS-FNAB) was performed to obtain a histological diagnosis preoperatively. The pathological findings showed collagenous stroma and inflammatory exudates with a small quantity of spindle cells under hematoxylin-eosin staining. Immunohistochemically, the spindle cells were positive for S-100, which pointed to a diagnosis of schwannoma.

**Figure 3:**
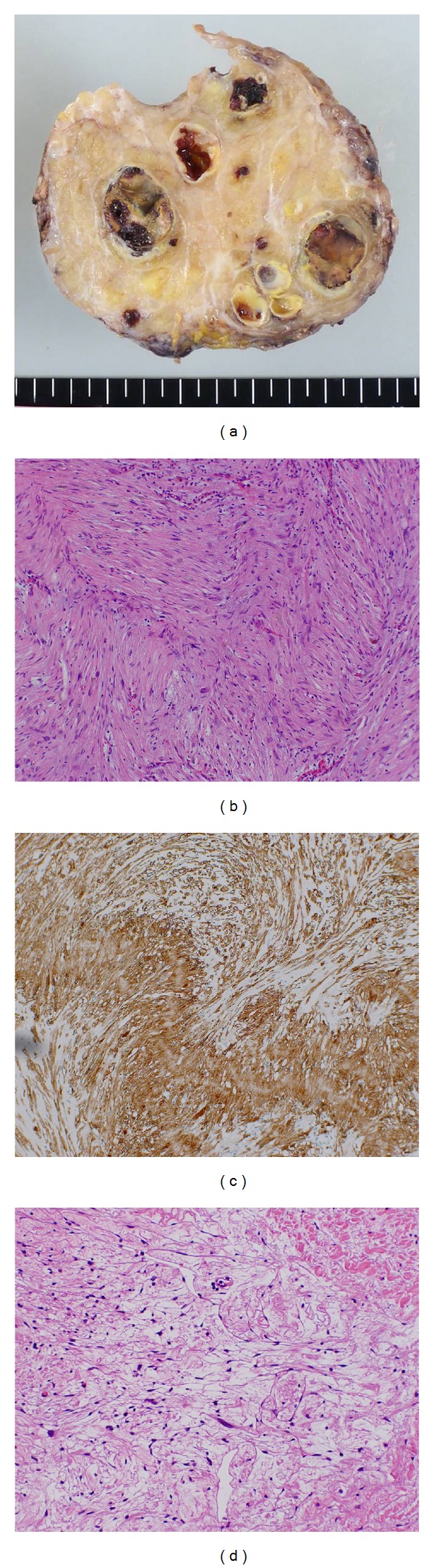
(a) Cut surface of the tumor shows encapsulated solid mass containing multiple eggshell-like calcifications. (b) Microscopic image (HE, magnification ×100) shows palisaded arrangement of spindle-shaped cells. (c) Immunohistochemically, tumor cells are positive for S-100 protein. (d) Myxoid degeneration and hyalinization are focally seen in the tumor.

Laparoscopic extirpation was carried out. The tumor was 110 mm on its major axis and was encapsulated. A cross section showed multiple spotted or oval calcifications. Histologically, sections showed the proliferation of spindle-shaped cells having wavy nuclei arranged in fascicular or palisading pattern. Myxoid degeneration, calcifications, and hyalinizations were also observed. Mitotic figures were inconspicuous. Immunohistochemically, tumor cells were positive for the S-100 protein. That feature indicated schwannoma, and there was no evidence of malignancy (Figure 3).

The patient's postoperative course was uneventful, and there were no signs of recurrence on physical or imaging examinations 6 months after the operation.

## 3. Discussion

Pelvic or retroperitoneal schwannomas commonly locate in the presacral region or lower retroperitoneum at the pelvic brim [2]. Preoperative diagnosis is difficult, however, because of its rarity and the nonspecific imaging features on CT and MR. Histologically, typical schwannomas are composed of intermixed Antoni A (compact cellular regions, arranged in short bundles or interlacing fascicles) and Antoni B components (loose and hypocellular regions with more myxoid or edematous components) [3].

At CT, a schwannoma typically appears as a well-demarcated round or oval mass, often showing prominent cystic degeneration and calcification. Calcifications are punctate, mottled, or curvilinear and are seen along the walls of the mass [4]. However, calcifications are uncommon in retroperitoneal schwannomas [1, 5]. Liu et al. mentioned that only one of eight retroperitoneal schwannomas exhibited calcifications [6]. At contrast-enhanced CT, schwannomas demonstrate variable homogeneous or heterogeneous enhancements. The heterogeneous enhancement represents variation in the degree of cellularity. Loose cellularity with diffuse edematous change may result in minimal contrast enhancement. Heterogeneous areas on enhanced CT may also be due to cystic and hemorrhagic changes [3, 7]. MRI findings in schwannomas have been described as masses with low signal intensity on T1-weighted image and heterogeneous high signal intensity on T2-weighted image due to alternating Antoni A and Antoni B areas and secondary degenerative changes. Contrast-enhanced T1-weighted images can clearly depict cystic areas and well-enhanced peripheral and intervening solid areas of such masses [3].

In our case, the tumor was a hypoattenuation pelvic mass with mottled or multiple oval, eggshell-like calcifications and was slightly enhanced at delayed phase on contrast-enhanced CT. The tumor demonstrated hypointensity and mild hyperintensity on T1-weighted image and T2-weighted image on MRI. Spotty areas of hyperintensity were also observed on T1-weighted image, which indicated hemorrhage. Typical cystic appearance was not evident on either CT or MRI. We speculated that the calcifications were a result of cystic degeneration or hemorrhage during their slow growth over a long period.

It is difficult to confirm the diagnosis of retroperitoneal schwannomas using only imaging examinations such as CT or MRI, in particular when the tumors do not reveal a typical solid and cystic appearance. As a result, pelvic schwannomas are often diagnosed by surgically resected specimens. CT- or US-guided FNAB constitutes a useful option for the diagnosis of pelvic schwannomas.

In conclusion, we describe a case of pelvic schwannoma with prominent calcifications. When diagnosing tumors, even those without typical solid and cystic appearances, the possibility of schwannoma should be considered. EUS-FNAB is also recommended in such difficult cases.
